# Geometrical analysis for assessing torsional alignment of humerus

**DOI:** 10.1186/s12891-020-3118-7

**Published:** 2020-02-10

**Authors:** Yo-Lun Chu, Cheng-Kuang Chen, Yu-Chia Liu, Tung-Wu Lu, Chen-Kun Liaw

**Affiliations:** 1 Institute of Biomedical Engineering, National Taiwan University, Taipei, 100 Taiwan; 2 Department of Orthopaedics, Taipei Municipal Wanfang Hospital, Taipei, 11696 Taiwan; 3 Department of Orthopedic Surgery, Shin Kong Wu Ho-Su Memorial Hospital, Taipei, 11101 Taiwan; 4 Department of Orthopaedics, Shuang Ho Hospital, Taipei Medical University, New Taipei City, 23561 Taiwan; 5 Department of Orthopaedics, School of Medicine, College of Medicine, Taipei Medical University, Taipei City, 11031 Taiwan; 6 Graduate Institute of Biomedical Optomechatronics, College of Biomedical Engineering, Research Center of Biomedical Device, Taipei Medical University, Taipei City, 11301 Taiwan

## Abstract

**Background:**

Compared to other types of surgeries, minimally invasive surgeries (MISs) of humeral shaft fractures are associated with less radial nerve injury, less soft tissue injury and higher union rate. However, malrotation often occurs in MISs when closed reduction methods are used.

This study aims to define specific palpable landmarks to help surgeons determine the correct torsional angle and reduce the incidence of malrotation.

**Methods:**

Twenty-eight normal humeral computed tomography scans were retrieved from our image database. One line was drawn through the vertices of the intertubercular sulcus of the humeral head in the coronal view, and another line was drawn through the longest axis between the medial and lateral condyles in the coronal view. The angle between these two lines was measured at least 3 times for each scan.

**Results:**

The profile of the intertubercular sulcus tangent line of the humeral head and the axis of the distal humerus was identified as the most accurate method for assessing the precision of torsion during MIS for humeral shaft fractures. The transepicondylar axis line is more internally rotated than the intertubercular sulcus tangent line. The mean angle was measured to be 41.1 degrees.

**Conclusions:**

The axis of the distal humeral condyles is internally rotated by approximately 41.1 degrees compared with the intertubercular sulcus tangent line of the humeral head. Minimally invasive surgeries can be performed by using these palpable landmarks. The torsional deformities can be reduced with the proper angle adjustment without the need for fluoroscopy. It can also be used to treat unstable comminuted humeral fractures.

**Level of evidence:**

Retrospective Study, Diagnostic study, Level III.

## Background

Fractures of the humeral shaft are common, as they account for 10% of long-bone fractures and 3–5% of all fractures [1, 2]. Humeral shaft fractures result in a significant burden to society due to lost productivity and wages. In the United States, over 66,000 cases occur annually and account for more than 363,000 days of hospital stays [3]. Both the incidence of humeral shaft fractures and the utilization of surgical interventions have been increasing over time [4]. A bimodal age distribution with one peak was found in men in their thirties, and another peak was found in women in their seventies [5]. Among them, the younger patients were included in the high-activity groups. For those with economic responsibility for the household, the recovery of upper limb function is very important.

The goal of treatment for humeral shaft fractures is bony union with an acceptable humeral alignment and a return to the pre-injury level of activity [6]. There is a high risk for pseudoarthrosis, as it occurs in as many as 29% of cases without surgical treatments [2, 7].

Among the many kinds of surgeries that are available for humeral shaft fractures, minimally invasive surgeries (MISs), which were first described by Livani and Belangero [8], are particularly important. MISs have been widely used for reduction and fixation of humeral shaft fractures with good results, as the biology and vascularization of fragments have been maximally preserved. Other advantages include the absence of injury to soft tissue, which leads to maintained blood supply to the bone, less blood loss, a shorter operative time [8–13], the best aesthetic result, and a lower rate of complications, such as non-union, radial nerve palsy, and infection [14–16].

The two main minimally invasive surgical techniques for fracture fixation are intramedullary (IM) nailing and minimally invasive plate osteosynthesis (MIPO). Indirect reduction techniques that do not reveal the site of fracture are always used during MISs [17]. Regardless of whether IM nailing or MIPO is performed, a reduction is usually evaluated using fluoroscopy. It is easy to identify valgus or varus deformities, but it is difficult to identify torsional deformities. Torsional deformities of various degrees may occur. Postoperative malrotation exceeding 20° was found in 40.9% of a group of patients who underwent MIPO [15]. The degree of malrotation correlates with a decreased range of motion and may be a cause of degenerative arthritis in the long-term [18].

Clinically, torsional alignment requires a surgeon’s clinical judgement under intraoperative fluoroscopy as he or she observes the shape of bone fragments or compares the affected humerus with the contralateral side [13]. Not only are patients and staff members in the operating room exposed to radiation, but the operation also takes longer to perform. For pregnant patients, avoiding the use of fluoroscopy during surgery is important. Moreover, for hospitals not equipped with fluoroscopy tools, surgeons may have difficulty identifying humeral deformities in the fracture via the MIS approach.

The angle of humeral head retroversion has been most commonly used for measuring the torsional degrees of the humerus [19–21]. However, when using humeral head retroversion as a torsional reference for the surgical treatment of humeral shaft fractures, fluoroscopic assistance or extensive operative wounds for exposure of the humeral head are usually required.

This study aims to define specific palpable landmarks to help surgeons measure humeral torsion and reduce the incidence of torsional deformities during MIS without fluoroscopic assistance. To the best of our knowledge, this is the first study assessing torsional alignment by examining the specific angle of humeral anatomic torsion using computed tomography (CT) images.

## Methods

### Studied population

All humeral CT scans between February, 2007 and August, 2018 were retrospectively retrieved from the Picture Archiving and Communication System (PACS) at our hospital. The images were selected for analysis according to the following inclusion criteria: (1) age between 18 and 90 years, (2) normal humeral structure without any congenital or acquired deformities, and (3) serial computed tomography records of the whole humerus.

Overall, the studied population comprised 28 paired cases, including 13 women and 15 men with a mean age of 54.9 years (range 18–89 years, median age 53 years).

This study was approved by the institutional review board (IRB) of Shin Kong Wu Ho-Su Memorial Hospital under IRB number: 20180809R, and was conducted in accordance with the relevant guidelines and regulations.

### Determination of reference landmarks

CT scans of the full-length humerus with a 5-mm slice thickness were performed to study the cross-sectional morphology and to identify landmarks for measurement.

**Fig. 1:**
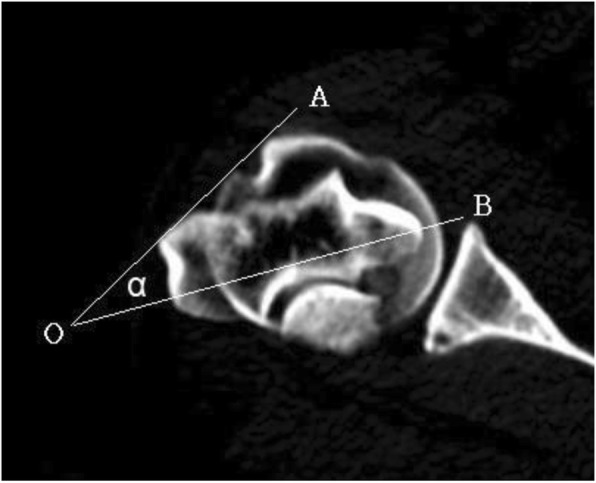
Two reference axis lines: OA line is the tangent line of the intertubercular sulcus; OB line is the transepicondylar axis line

**Fig. 2:**
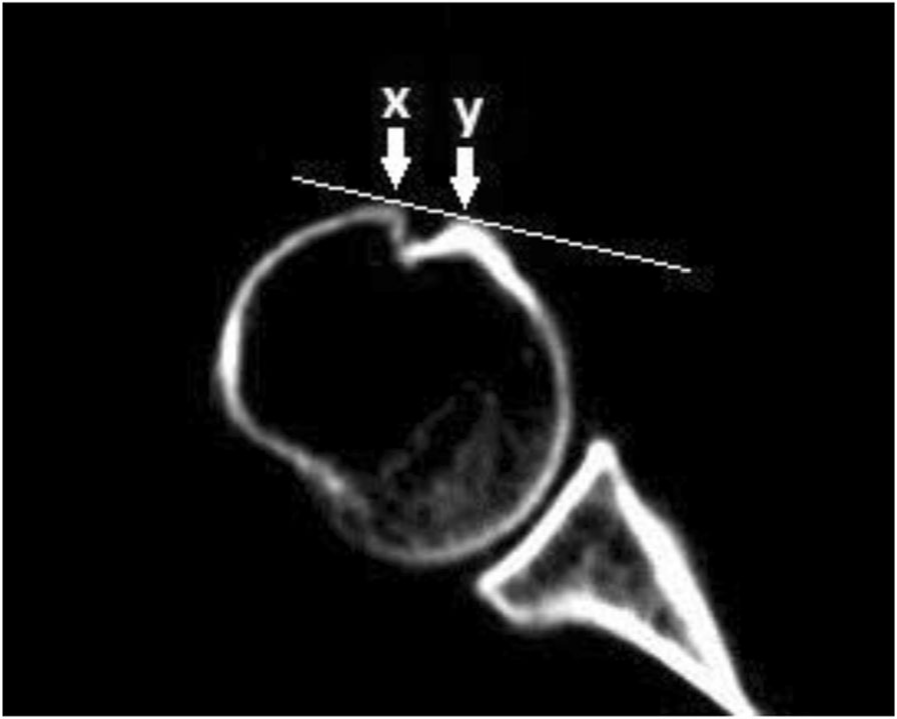
Points x and y are the vertices of biceps groove. The two points are connected in a tangent line of the deepest intertubercular sulcus

The proximal orientation of the humerus was first measured on the section of the deepest intertubercular sulcus. One line (OA line in Fig. 1) was drawn through the vertices of the deepest intertubercular sulcus (points x and y in Fig. 2) of the humeral head in the coronal view. Another line (OB line in Fig. 1) was drawn through the longest axis between the medial and lateral condyle in coronal view. The angle (called the “α” angle in this study) between the tangent line of the intertubercular sulcus and the transepicondylar axis line was identified.

### Statistical analysis

Each measurement was conducted at least three times by the same orthopaedist with 8 years of institutional experience and the average value was used for analysis.

All statistical analyses were performed using IBM SPSS Statistics Subscription version 1.0.0.1174.

## Results

We collected demographic and measurement data for all 28 subjects (Table 1). The “α” angle was correctly identified in all subjects.

**Table 1:** Demographic and measurement data

Patient	Humerus (cm)	α angle(∘)	z-scores
1	26.36	55.6	0.8
2	28.60	59.5	1.1
3	28.88	23.8	−1
4	31.74	40.9	0
5	26.87	30.6	−0.6
6	25.62	45.6	0.3
7	29.86	21.2	−1.2
8	31.57	33.0	−0.5
9	26.40	48.3	0.4
10	28.50	37.6	−0.2
11	27.72	51.8	0.6
12	28.02	38.8	−0.1
13	27.96	65.1	1.4
14	31.81	40.1	−0.1
15	30.77	41.6	0
16	32.04	60.4	1.1
17	28.88	10.7	−1.8
18	27.70	38.2	−0.2
19	31.57	73.1	1.9
20	31.00	1.0	−2.3
21	30.82	25.1	−0.9
22	28.36	37.9	−0.2
23	30.60	26.2	−0.9
24	29.33	48.8	0.4
25	31.41	65.1	1.4
26	28.40	31.5	−0.6
27	32.49	64.4	1.4
28	28.43	34.8	−0.4

The transepicondylar axis line was more internally rotated than the connecting line at the vertices of the intertubercular sulcus. As shown in Table 2, the mean angle was measured to be 41.1° (range 1.0°-73.1°), with a standard deviation of 17.1°. The outliers were determined by calculating the z-score, and z-scores greater than 2 or less than − 2 were considered outliers. There was only one outlier with an α angle of 1.0°. When the outlier was excluded, the mean angle was 42.6° (range 10.7°-73.1°), and the standard deviation was 17.1° (Table 2).

**Table 2:** Clinical validation results on α angle

Outlier	Minimum	Maximum	Mean	Standard deviation
With	1.0∘	73.1∘	41.1∘	17.1∘
Without	10.7∘	73.1∘	42.6∘	15.5∘

Table 3 shows the correlations of the α angle with the humeral length and patient age. The correlation coefficient between the α angle and humeral length was 0.42, while that between the α angle and age was 0.52.

**Table 3:** Correlation of α angle with humeral length and age

Variables	Pearson product-moment correlation coefficient	Degree of correlation
α angle & Humeral length	0.42	Low
α angle & Age	0.52	Moderate

The intraclass correlation coefficient (ICC) with a two-way random effects model of consistency was used to analyse measurement reliability. For the measurement of the α angle, the intraobserver correlation was excellent (Table 4).

**Table 4:** Intraclass correlation coefficients (ICC) in the intraobserver measurement

Intraobserver reliability	Single measures	Average measures
ICC	0.974	0.991

## Discussion

The purpose of this study was to determine the landmarks important for evaluating torsional deviations of the humerus when minimally invasive surgical techniques are used for humeral fracture fixation. The results obtained showed that differences in the angle between the transepicondylar axis line and the connecting line at the vertices of the intertubercular sulcus ranged from 1.0° to 73.1°, with a mean angle of 41.1°. This study identified the profile of the intertubercular sulcus line of the humeral head and the axis of the distal humerus as an accurate tool for assessing the precision of torsion during MISs for humeral shaft fractures without fluoroscopic assistance. The line drawn through the vertices of the intertubercular sulcus line of the humeral head was externally rotated by approximately 41.1° compared with the axis of the distal humeral condyles.

In practice, surgeons may palpate the bony prominence of the intertubercular sulcus of the proximal humeral fragment as a landmark to evaluate the tangent line. Surgeons may rotate the distal humeral fragment, making the transepicondylar axis line internally rotated by 41.1° compared with the tangent line of the intertubercular sulcus. After the fixation is completed and stable, a clinical exam of internal rotation and external rotation of the shoulder joint can be performed to determine whether the forearm is oriented in the proper direction [22]. Thus, the fracture will undergo proper torsional reduction. Additionally, the technique may be effective for unstable comminuted or segmental humeral fractures (AO type 12C) if it is difficult to maintain the fracture in adequate alignment during the operation. A clinical trial should be conducted in the future as a final test. This technique needs to be demonstrated to prevent an increase the numbers of torsional malalignments and complications.

If a preoperative CT scan is available, the fracture can be virtually reduced using 3D system software [23]. The reconstructed model can be used to restore the physiological magnitude of humeral torsion and measure the alpha angle of the humerus.

In this study, the parameters of the humerus were measured using CT. In all 28 cases, the connecting line at the two vertices of the intertubercular sulcus was found to be externally rotated compared with the transepicondylar axis line.

Measurements were made on serial images of the humerus with 5 mm between each section. Errors may occur because the deepest sulcus and the longest axis of condyles of the sections taken for measurement may not be the actual deepest and longest ones.

Previous research studies considered a humeral malrotation of 15° in fracture alignment acceptable [24]. Although the standard deviation of the α angle obtained in this study was 17.1°, the mean angle may still be used as a reference for reduction. Statistically, an extreme outlier in the present data was identified. The standard deviation without the outlier was 15.5°, which is close to the abovementioned acceptable degree (15°) of humeral malrotation.

The proximal incision during MIPO is made with the deltopectoral approach [25]. The surgeon can directly touch the biceps sulcus as a landmark. The soft tissue around the elbow is thin in most cases. Epicondyles can be easily touched to identify the transepicondylar axis. The axis identified by the surgeon is not very different from the actual direction.

The palpable proximal and distal osseous landmarks (intertubercular sulcus, medial and lateral epicondyles) located by orthopaedic surgeons are slightly different from the imaging landmarks. For example, the biceps groove is located at the proximal humerus and becomes shallower toward the inferior end. The proximal landmark over the intertubercular sulcus in this study was obtained from the deepest site. Surgeons may not be able to locate the deepest point of the bicipital groove. However, extensive surgical experience and good judgement can increase the accuracy of identifying this groove. Highly trained and specialized orthopaedic surgeons can precisely locate the deepest groove and other anatomical landmarks.

However, the landmarks are not applicable in some situations, such as when the humeral head or the distal humerus is severely deformed due to acquired or congenital disorders.

In past research, a strong relationship has been shown to exist between humeral torsion variables obtained with ultrasound and CT [26]. If ultrasound equipment is available in the operating room, an ultrasonographic assessment of the humeral retroversion method can be used as a secondary confirmation [22]. In addition, when the patient’s soft tissue layer is thick and it is difficult to palpate the bony landmarks, ultrasonic positioning can be used to identify the transepicondylar axis.

Various techniques have been employed to measure torsional parameters of the humeral bone. Retroversion of the humeral head is most commonly used for defining the angular difference between the orientation of the proximal humeral head and the axis of the elbow at the distal humerus [19]. However, the results are highly variable, ranging in some case series from − 6° to 50° [27–30].

Nevertheless, obtaining the retroversion angle in operation requires fluoroscopic assistance, and it is difficult to confirm whether the proximal line is perpendicular to the articular surface.

A previous study used the bicipital groove of the humeral head to predict the torsional state of the humerus for intraoperative evaluations, but fluoroscopy was still needed with this approach [31].

To the best of our knowledge, no other study has used CT to measure humeral torsion with the tangent line of the intertubercular sulcus. A similar study measured humeral head retroversion with lateralization of the intertubercular groove using CT [32], which seems to be valuable for anatomical imaging but unsuitable for clinical orthopaedic surgeries.

This study also identified the correlations of the α angle with the humeral length and patient age. The correlation between the α angle and humeral length was statistically low, while that between the α angle and patient age was moderate. In the absence of a strong correlation with the α angle, age and humeral length need not be considered in clinical-surgical evaluations.

There were 13 right and 15 left humerus bones included in the research study. The mean alpha angle in the present study was 37.4 ± 18.6 degrees on the right side and 44.3 ± 15.7 degrees on the left side. The sample size is small, so the statistical significance of the results is unclear.

Previous studies have shown that the dominant arm of patients has a higher retroversion angle than the contralateral arm. On average, the degree of retroversion is 10.6 degrees larger in the dominant arm compared with the nondominant arm in overhead throwing athletes [33, 34]. Although the alpha angle is not an exact measure of humeral head retroversion, perhaps the angles on the bilateral humerus are different for people who have participated in throwing sports. However, whether the participants in our study practised throwing sports was not recorded. In a future study, we can add this factor to determine whether it has statistical significance.

Finally, only 28 extremities from 28 participants were included in the analysis. The volume of data in our imaging system limited our sample size. While more patients should be included in future prospective research studies, the costs and radiation exposure associated with CT scans should be taken into consideration when designing these studies.

## Conclusions

The intertubercular sulcus and humeral condyles are easy to identify by palpation and are useful landmarks. Compared with the tangent line of the intertubercular sulcus, the transcondylar axis is internally rotated by 41.1 degrees. The alpha angle can be effectively used in minimally invasive surgeries or unstable comminuted fractures to reduce torsional malalignment without fluoroscopic assistance. However, additional clinical studies are necessary to further verify these conclusions.

## Data Availability

The datasets used and analysed during the current study are available from the corresponding author on reasonable request.

## Abbreviations

AO: Arbeitsgemeinschaft für Osteosynthesefragen (German for “Association for the Study of Internal Fixation”)
CT: Computed tomography
ICC: Intraclass correlation coefficients (ICC)
IM: Intramedullary
IRB: Institutional review board
MIPO: Minimal invasive plate osteosynthesis
MIS: Minimally invasive surgeries
ORIF: Open reduction internal fixation
PACS: Picture Archiving and Communication System
